# Death and Mourning Process in Frontline Health Care Professionals and Their Families During COVID-19

**DOI:** 10.3389/fpsyt.2021.624428

**Published:** 2021-03-22

**Authors:** Sreeja Das, Tushar Singh, Rahul Varma, Yogesh Kumar Arya

**Affiliations:** Department of Psychology, Banaras Hindu University, Varanasi, India

**Keywords:** COVID - 19, death, mourning, frontline health care professionals, grief, psychosocial support

## Abstract

The COVID-19 epidemic has mushroomed globally, disrupting the existence of millions. Under this current pandemic situation, the frontline health care professionals are looped in the clutch of the virus and are relatively more exposed to the patients infected with the disease. In this precarious situation, the frontline health care professionals have contributed their best to provide utmost care to the patients infected with the ailment. The direct involvement of these professionals, however, has taken a toll on their physical health as well as on their mental well-being. Several studies conducted recently have reported that frontline health care workers engaged in direct diagnosis, treatment, and care of patients with COVID-19 are associated with a higher risk of symptoms of depression, post-traumatic stress disorder and other mental health issues. Lack of personal protection equipment, unreasonable amounts of work, improper medicines, fear of contracting the disease, and lack of skilled training have interposed the frontline health care workers with unimaginable stress. Due to the widespread outbreak, the death count of the frontline health care professionals has also surged. However, studies exploring the physical and mental welfare of the frontline health care professionals and their families are very few and far behind. To address this aperture, the present paper attempts to highlight the psychological and physical impact of the COVID-19 pandemic on the frontline health care professions and to understand the impact of the death of these frontline health care professionals on the psychological well-being, mourning process, and complicated grief among the family members of healthcare professionals. The paper also presents some recommendations for providing psychological support to healthcare professionals and their bereaved families.

## Introduction

Due to its unprecedented and unforeseeable nature, the COVID-19 virus epidemic has plunged the entire world into an indefinite zone. During this time of predicament, immense pressure has been donned upon the frontline health care professionals (1) who provide necessary services to the populations. They include doctors, nurses, midwives, and pharmacists (2) working in the health sector. Shanafelt et al. (3) have noted that the exponential rise of COVID-19 and the severity of symptoms have taxed the boundary of health industry. They further outlined eight major sources of anxiety during this pandemic on frontline health care professionals. These are (1) difficulty getting the appropriate amount of personal protective equipment, (2) exposed to vulnerable places and fear of contracting the virus, (3) less access to proper testing for COVID-19 and fear of spreading disease to co-workers and family, (4) uncertainty of who will take care of the family if they contract the virus (5) giving time to their wards and spouses amidst lockdown of work and schools, (6) availability of essential commodities for (e.g., food, water, lodging, logistics), (7) being able to be efficient and provide health care if positioned in a new place and (4) lack of updated information.

During the unprecedented times, health care professionals and non-health care professionals have lost their lives in an unexpected manner. World Health Organization (WHO) have reported that ~14% of the frontline health care professionals have been infected with COVID-19 and that in the case of countries falling in low- and middle-income strata, this can be as high as 35%. Furthermore, along with the increase of physical hazards due to the virus, the pandemic has also put a bizarre burden of psychological health issues on health care professionals which include, but are not limited to, somatization, obsession, compulsion, anxiety, phobic anxiety, and psychoticism (5). These physical and psychological sufferings among health care professionals have led to the development of constant fear of contracting the disease and taking it back to the family. This becomes more prominent as the COVID-19 pandemic has upraised a novel problem with regards to the clinical methods of conducting cardiopulmonary resuscitation (CPR) with utmost clinical protection (6) and thus health care professionals deployed in emergency care units are more prone to be infected with this disease.

The present paper aims to highlight the psychological and physical impact of the COVID-19 pandemic on the health care professionals and to understand the impact of the death of these frontline health care professionals on the psychological well-being, mourning process, and complicated grief among their family members. The paper also provides some recommendations for providing psychological support to healthcare professionals and their bereaved families.

## Impact on Physical and Mental Health

Jahrami et al. (7) have testified that 3/4th of healthcare workers in COVID-19 care centers have reported disturbances in sleep cycle due to the constant work stress, dealing with death and dying of patients and co-workers, chaotic work schedule and work cycle. Recent studies have established that due to the close contact with patients diagnosed with COVID-19, the frontline health care professionals have developed physical symptoms like fever, headache, cough, hemoptysis, and diarrhea (8, 9). In other studies, skin disease, tightness of skin, nasal bridge, and dryness have been observed in the health care workers who claimed to have worn PPE kits, surgical gloves, and face shields for a long number of hours (9–11). Xiang et al. (12) also reported that frontline health care professionals, especially who are deployed in hospitals as a caregiver of the infected patients, have more plausible chances of contracting the disease.

Earlier studies in epidemic contexts have reported that among the health workers in high-risk clinical settings such as SARS units in Beijing hospital, those with a family history of same illness had more bouts of traumatic flashbacks and more post-traumatic stress symptoms than those without family history. Healthcare professionals working with COVID-19 patients have been shown to display mental health issues such as depression, anxiety, insomnia, and distress (9). Cabarkapa et al. (13) conducted a systemic review of available literature and concluded that frontline nurses frequently report somatization, sleep disorders and insomnia. In addition, they also reported that female nurses who are in close contact with COVID-19 patients have higher mental health issues. Similarly, Kang et al. (14) have reported that about 34.4% of healthcare staff show mild mental health issues and 6.2% have shown severe mental health issues. Studies have also reported burnout and high levels of fear among the healthcare staff (15).

## Impact of Death of Frontline Health Care Professionals on Their Family

Due to the unprecedented nature of the pandemic, most countries could not restrict the surge of cases, thus leading to a massive outbreak. At the onset, the workers were devoid of proper training to deal with the unaddressed situation, with a lack of adequate equipment and drugs. Yoshida et al. (16) reported that the spout of confirmed cases and delay in implementing lockdown overtaxed the medical staff with inadequate supplies. Healthcare workers' jobs in such a situation were in the context of high personal risk of becoming infected and their motivation to provide professional services were largely altruistic (17, 18). A large number of health care workers were infected with COVID-19 and a significant proportion among them lost their lives in almost all parts of the world. In September 2020, the Pan American Regional Office of the World Health Organization in Washington reported the death of 2500 health care staff due to COVID-19 (19). The COVID-19 infections and resultant death of health care staff is a major source of grief and other psychological problems for their families and colleagues as well as for local and national healthcare infrastructure (4). In one of the current studies, deaths of frontline health care workers due to COVID-19 has been shown to result in prolonged grief disorder, post-traumatic stress, and other poor bereavement outcomes among their relatives (20).

Holmes and Rahe (21) stated that the deaths of dear ones lead to physical and mental issues due to the loss incurred. Malkinson (22) reported in one of his studies that the coping mechanism of the members of the family depends on a numerous factors which include coping strategy, affiliation with the departed, and the reason for the death caused. Although in most cases the family copes with the bereavement without any grief counseling sessions, Prigerson et al. (23) testified that around 10% of the bereaved persons are vulnerable to complicated grief after the death of family members and 30% fall in the moderate mental health risks. Additionally, Aoun et al. (24) mentioned that the causal factors that delay the coping consequences may include medical records due to psychiatric issues, lack of environmental upkeep, or a precipitous death (25–27).

Dorothy et al. (28) conducted interviews with trauma survivors over the years and suggested that compared to the grief experienced due to interrupted loss, the multiple losses have a greater impact on the bereaved individuals. They reported that grief experienced by the victim's family is generally complex and lasts longer than expected.

In a pandemic situation, balancing work and life is a very exhausting and stressful task for the frontline health care workers. Therefore, it is very difficult for the spouses of the healthcare professionals to deal with the situation as they are in the constant fear of contracting disease. In the world where social media has taken over everything, and every trivial detail is broadcast on the electronic media, family members are living a life in the constant fear of losing their loved ones and preparing themselves for the irreplaceable losses. To add to their sufferings are their inability to attend burials, cremation, or bid final goodbye to their dear ones due to the contagious nature of the virus (29, 30).

## Recommendations for Helping the Mourning Process in Healthcare Workers' Families

The very first thing about which we can talk in the mourning process is the trauma without bereavement. In this process a traumatic event experienced by the person escalates traumatic symptoms which leads to the diagnosis of acute stress disorder or post-traumatic disorder, which are mostly determined by the time frame of the onset of the disorder. Symptoms related to anxiety and depression may also lead to a concomitant diagnosis. The second thing in the mourning process is the bereavement without trauma. Here the person experience traumatic events without the symptoms as they witness the demise of their beloved ones. If there are convolutions after the demise, one of the complex mourning categories would relate to this complication. The third thing is traumatic bereavement that includes few major things which are escalating traumatic symptoms. These are person's own experience of death (often occurring with an altercated relationship with the dead or an unstable attachment) or there is something related to death itself (often brutal deaths) or when the person encounters a death. Parkes (31–33) has explained the mourning process in terms of several phases. In the phase I person feels the numbness that happens nearly from the time of the demise. The numbness helps survivors to forget the loss at least for a short period of time. After that the person enters phase II, defined as period of yearning, in which the person yearns to get back the lost one and does not accept the irreversible loss. Anger acts as the most important part of this phase. In phase III, the period of anguish and chaos, at this point of moment, it is very difficult for the bereaved person to adjust to the environment. In the end, the bereaved person is able to enter phase IV, the period of reconstructed behavior, and begin to stabilize in their life. Bowlby (34), whose interest and work area coincide with those of Parkes, supported the notion of phases and put forward that the bereaved must undergo a similar sequence of phases before mourning is ultimately resolved. Sander (35, 36) also used the notion of phases in her study and reported five of them: (1) shock, (2) awareness of loss, (3) conservation withdrawal, (4) healing and (5) renewal.

The grief and morning processes are humans' natural responses to the deaths of their loved ones (37). Various cultural rituals such as traditional funeral and burial are meant to facilitate this mourning process among the deceased's family members. Death ceremonies play an important role in mourning, bringing together those who recall the dead person to honor their life, and providing an empathetic network for the bereaved family (29, 38). However, during the current COVID-19 pandemic the family members of the deceased healthcare workers were not able to attend and properly perform the funerals and burial of their departing family members. The pandemic interrupted the mourning activity by affecting families' ability to hold funerals and other ceremonies. Also, the much-needed social support is also not available to these members due to the practice of social distancing during COVID-19 pandemic. Thus, the improper grief responses and incomplete mourning process have put these families at the risk of mental health issues, prolonged grief and mourning process, as well as reduced quality of life (39).

## Recommendation to Help Bereaved Families of Healthcare Professionals

Under the current pandemic situation, due to social distancing norms and several restrictions, families and relatives of the deceased individuals are unable to come together to share their grief, care, and love with each other. This may lead the families of deceased health care providers to develop unresolved prolonged grief and complicated psychological reactions. Under such a situation a comprehensive support mechanism to provide support and counseling to the bereaved families is the need of the hour. Some of the strategies that could be adopted to help these bereaved families are recommended here.

Wallace et al. (29) have stated the major cause for not dealing with the complicated grief experienced is due to the fact that the family members are least prepared for the death of their loved ones. In such a situation, support groups and counseling sessions should be provided to the bereaved families along with the ‘clapping campaigns’ to reduce stress and overwhelming condition of the family members including spouses and children. Also, regarding the health care professionals, Government and non-government organizations must take responsibility to assure that healthcare staff and their families are prepared for the emotional outcomes of their work and that resources, training, and supervision are in place to safeguard health care providers' health (40). Frontline staff should be seriously monitored by the organizers, must experience effective team cohesion, and must be involved in the execution of the strategies to keep up the everyday tasks of the team, combined with unconventional debriefing and support from fellow workers. It must also be ensured that the necessary social and mental support are available to these individuals to help them better understand and accept the reality, deal with the resultant stress, re-organize their lives, and reduce their sufferings to compensate the natural mourning process (41). As Brooks et al. (42) suggest, ample amount of training, protection, and assistance could help to keep health care workers away from mental illness.

On the basis of their experiences of supporting 246 families of COVID-19 victims, Borghi et al. (43) have suggested some strategies that were successfully used by family members to deal with the extraordinary mourning process caused by COVID-19. These include organizing alternative good-bye rituals, readdressing their hope and faith, supporting others, and conveying the bad news to others.

The use of advanced information and communication technology such as smartphones, tablets and internet could also be promoted (a) to maintain virtual connections among family members and relatives, (b) to broadcast the traditional rituals and funerals to those members of the family who are not able to attend due to restrictions, and (c) to organize post funeral activities (i.e., commemoration), care and sharing among members of family as well as those in the immediate support system.

## Conclusion

COVID-19 has left the frontline health care professionals with health uncertainty. The virus has donned upon the frontline health care professionals to a larger extent leaving a deeper impact on their physical and psychological well-being. Forefront medical workers and researchers have played a foremost role in combating against the COVID-19 outbreak (44). The present paper has outlined the stressors which the frontline health care professionals have faced including fear of spreading disease to co-workers and members of the family, irregular sleep patterns, abrupt work cycles, and lack of adequate training skills in dealing with such a novel situation. Many recent studies have highlighted the psychological issues which has impacted the lives of the frontline health care professionals (i.e. depression, anxiety, insomnia (45), and post-traumatic stress symptoms) (46). There are rare evidences of studies that have explored the mourning processes or complicated grief faced by the families of these health care professionals who lost their life fighting the pandemic. The paper has also attempted to make some recommendations to provide psychological support to the bereaved family, to fight the mental health problems encountered after the loss of their loved ones.

## Limitations and Further Suggestions

The present paper is an attempt to put forward the psychological problems being faced by frontline healthcare providers and their families amidst the COVID-19 pandemic. However, the present article has some limitations. The present paper lacks the empirical evidence, in the context of COVID-19 pandemic, to support the arguments made. This paper, therefore, calls for the planning and conduction of empirical investigations to understand the issues being faced by the family members of the deceased healthcare professional and to empirically test the effectiveness of the support strategies suggested in this paper. Studies are also needed to understand the changes that have been brought about by this pandemic into the grief and mourning process of the deceased family members and to find strategies to improve the existing support systems so as to address the changes brought about by the present as well as future pandemics.

## Data Availability Statement

The original contributions presented in the study are included in the article/Supplementary Material, further inquiries can be directed to the corresponding author/s.

## Author Contributions

SD and TS conceptualized the manuscript theme. SD wrote the first draft. RV and YA reviewed and improvised the draft. SD and TS prepared the final manuscript draft for submission.

## Conflict of Interest

The authors declare that the research was conducted in the absence of any commercial or financial relationships that could be construed as a potential conflict of interest.
